# Fibrous dysplasia: A tale of two syndromes

**DOI:** 10.4102/sajr.v28i1.2877

**Published:** 2024-05-23

**Authors:** Jacques Fourie, Farhana Suleman, Zarina Lockhat, Kumeshnie Kollapen

**Affiliations:** 1 Department of Radiology, Faculty of Health Sciences, University of Pretoria, Pretoria, South Africa; 2 Department of Radiology, Faculty of Health Sciences, Steve Biko Academic Hospital, Pretoria, South Africa; 3 Department of Radiology, Faculty of Health Sciences, Kalafong Provincial Tertiary Hospital, Pretoria, South Africa

**Keywords:** fibrous dysplasia, Mazabraud syndrome, McCune–Albright syndrome, musculoskeletal, skeletal dysplasia

## Abstract

Fibrous dysplasia (FD) is a rare, non-inherited, congenital bone disorder which may be monostotic or polyostotic. The polyostotic form may rarely present in syndromic forms when associated with extra-skeletal manifestations. Mazabraud syndrome is a rare syndrome consisting of polyostotic FD presenting with intramuscular myxomas. McCune–Albright syndrome is recognised by polyostotic FD, precocious puberty and ‘café au lait’ spots. This report describes an adult patient with Mazabraud syndrome and a child with McCune–Albright syndrome.

**Contribution:**

Radiographic findings are typical with bowing deformities, sclerotic, lucent or mixed lesions and bony expansion, often with endosteal scalloping. MRI is often non-contributory and may actually mimic a more aggressive process. Early detection and correct diagnosis allow for early preventative treatment and rehabilitation to prevent devastating neurological sequelae and disability.

## Introduction

Fibrous dysplasia (FD) is a rare, non-inherited, congenital bone disorder involving the developmental and proliferative process of bone formation. The disease may be monostotic or polyostotic with the monostotic type being six times more common. The polyostotic form may rarely present in syndromic forms when associated with extra-skeletal manifestations. Mazabraud syndrome is a very rare syndrome consisting of polyostotic FD, presenting with intramuscular myxomas. McCune–Albright syndrome (MAS) is recognised by polyostotic FD, precocious puberty and ‘café au lait’spots.

Fibrous dysplasia results from a genetic mutation of the G stimulatory protein. Normal bone matrix and marrow is replaced by disorganised fibro-osseus tissue, which can involve single (Monostotic FD) to multiple bones (polyostotic FD).^1^ It accounts for approximately 7% of all benign bone tumours. McCune–Albright syndrome and Mazabraud syndrome (MS) are rare entities within the polyostotic FD spectrum with MAS accounting for less than 5% of polyostotic FD. Only around 100 cases of MS have been reported up to 2019.^2,3^

This report describes a case series of syndromic patients with polyostotic FD with emphasis on the radiological findings and a review of the literature.^4^

## Case 1

**FIGURE 1:**
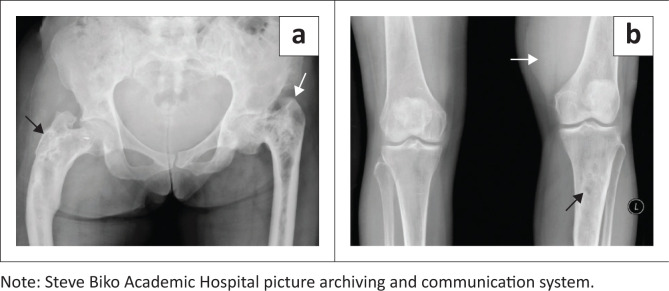
Anterior-posterior (AP) radiograph of the pelvis (a) demonstrates mixed lytic, sclerotic, expansile lesions (white arrow) with associated varus angulation and bowing, and shepherd crook deformity of the proximal femur (black arrow). AP radiograph of the knees (b) shows a similar lesion within the left proximal tibia (black arrow) with a soft tissue mass adjacent to the left medial distal femur (white arrow).

A 54-year-old woman presented to the orthopaedic tumour clinic at our institution with a history of a slow-growing, painful, soft tissue mass in the distal left thigh. The patient was admitted for further work-up. The biochemical studies were normal. Radiographs of the pelvis (Figure 1a) demonstrated expansile, mixed lytic and sclerotic lesions of both proximal femora with an associated shepherd crook deformity of the right femur and cranial migration of the left femur. Radiographs of the knees (Figure 1b) showed a mixed lytic-sclerotic lesion of the left proximal tibia with an impression of a soft tissue mass adjacent to the medial distal femur. The bony findings were in keeping with polyostotic FD.

**FIGURE 2:**
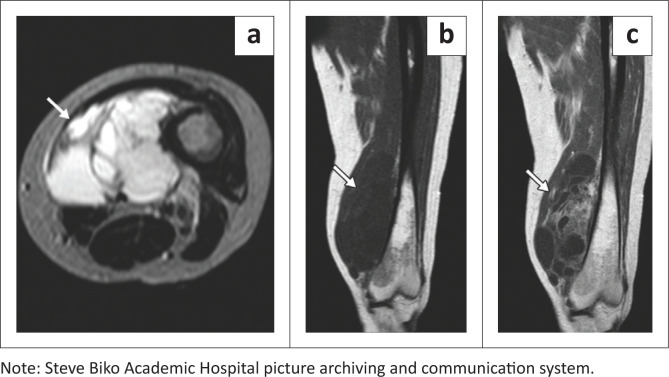
Axial T2-weighted images (a) of the left thigh confirm the intramuscular myxomas, which are T2 hyperintense with internal septations. Coronal T1 pre- and post-gadolinium images (b, c) demonstrate hypointense myxomas with heterogenous peripheral and patchy internal enhancement. The lesions are within the vastus medialis muscle.

An MRI scan of the left femur demonstrated a septated intramuscular mass hyperintense on T2-weighted imaging (T2WI) (Figure 2a) and hypointense to muscle on T1-weighted imaging (T1WI) (Figure 2b) with inhomogeneous contrast enhancement (Figure 2c). The MRI findings suggested a benign lesion characterised by the lack of intense contrast enhancement together with a more homogenous T1-weighted appearance and no intralesional fat, as seen in myxoid liposarcoma. The presence of higher apparent diffusion coefficient values is useful in distinguishing myxoma from myxoid liposarcoma with values less than 2.06 × 10−3 mm^2^/s supporting the latter. However, ADC and diffusion weighted sequences were not performed in this case.^5^ These findings were suggestive of a myxoma.

**FIGURE 3:**
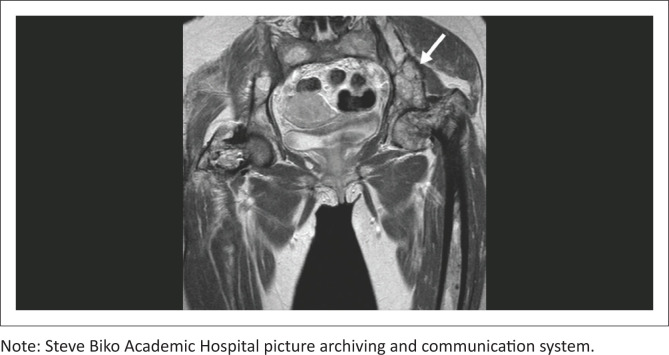
Coronal T1 post-gadolinium image of the pelvis confirms multifocal, enhancing, intra-osseous lesions within the left ilium (white arrow), right ilium, sacrum and proximal femora.

The pelvic MRI (Figure 3) demonstrated expansile, enhancing lesions within the left iliac wing and the proximal femora bilaterally. Further lesions were observed in the sacrum, vertebral bodies and left distal femur (images not shown). Investigation with excisional biopsy of the left thigh mass revealed a hypocellular, spindle cell lesion with abundant myxoid stroma suggestive of an intramuscular myxoma. The combination of clinical, radiological and pathological findings confirmed a diagnosis of MS. Management of the patient was symptomatic and follow-up was scheduled for evaluation of recurrence.

## Case 2

A 5-year-old girl presented to the orthopaedic clinic with a painful hip and facial dysmorphism. On examination, the clinician noticed that the patient was immobile, the right leg was externally rotated with a leg length discrepancy and there was tenderness over the femur well as features of precocious puberty. The patient was assessed as a Tanner stage 2 based on clinical examination.

**FIGURE 4:**
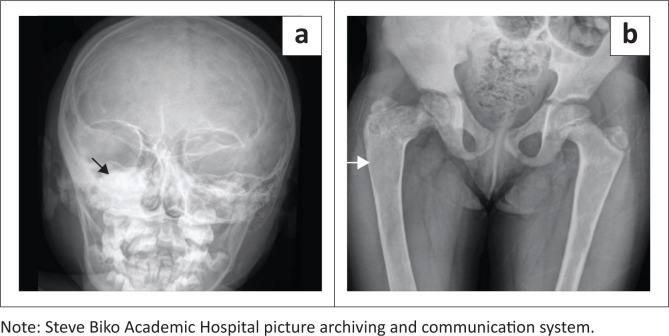
Anterior-posterior (AP) radiograph of the skull (a) demonstrates a unilateral, expansile sclerotic lesion of the spheno-temporal bone (black arrow). AP radiograph of the pelvis and proximal femora (b) demonstrate an expansile, mixed, lytic-sclerotic lesion of the right proximal femur (white arrow) with endosteal scalloping.

Radiographs demonstrated features of polyostotic FD with an expansile and predominantly sclerotic lesion in the right sphenoid bone (Figure 4a). The right femur demonstrated mixed lytic-sclerotic changes with a shepherd crook deformity (Figure 4b). There was also endosteal scalloping and expansion of the left proximal femur.

**FIGURE 5:**
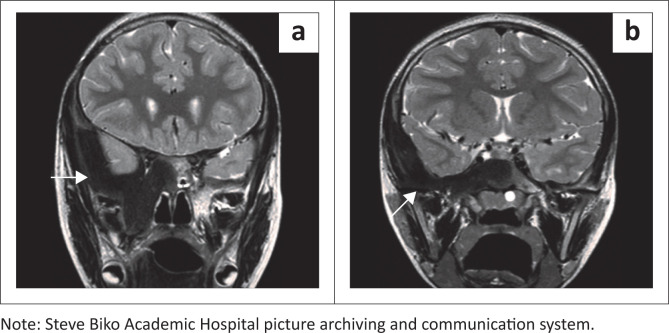
Coronal T2-weighted MRI images (a, b) confirm focal unilateral involvement of the spheno-temporal bone with homogenous hypointensity (white arrows). Associated extension into the pituitary fossa is demonstrated with minimal cranial displacement of the hypothalamic-pituitary axis. The hypothalamic-pituitary axis was otherwise normal.

An MRI scan of the brain was requested to exclude a pituitary tumour as a cause for the precocious puberty. The MRI revealed expansion of the right sphenoid and temporal bones (Figure 5a and b) with low-intensity signal on all sequences in keeping with the sclerosis noticed on plain radiographs. The hypothalamic-pituitary axis was normal and a diagnosis of MAS was confirmed. The patient was treated conservatively with regular endocrinology and orthopaedic follow-up.

## Discussion

Fibrous dysplasia is a benign, non-inherited, developmental bony condition. It consists largely of normal bone replaced by fibrous stroma and immature bone because of abnormal osteoblast differentiation. The disease pattern is defined by the number of bones affected, namely, monostotic, involving only a single bone or polyostotic, involving more than one bone.^3^ The incidence of FD is estimated to range between 1:5000 and 1:10 000 and accounts for approximately 5% of all benign bone tumours.

Monostotic FD is generally asymptomatic and is most prevalent in patients between the ages of 10 years and 30 years. It accounts for approximately 80% of all FD cases without gender predilection. Polyostotic FD presents at a younger age with no gender predilection and affects predominantly the craniofacial bones, ribs and long bones. A small percentage of patients with polyostotic FD have associated endocrine abnormalities. Monostotic FD is more prevalent than the polyostotic form with a ratio of 7:3.^4,6^ Fibrous dysplasia may be complicated by pathological fractures or associated aneurysmal bone cysts.^5^ Malignant transformation is thought to occur in less than 5% of cases of FD.^6^ With FD and MS, the aetiology appears to be gene related with the majority of the patients showing guanine nucleotide binding protein, alpha stimulating (GNAS) mutations at different stages of cell maturity, affecting uncontrolled cell proliferation with the pathogenesis of FD and MS providing different clinical presentations with varying degrees of pain and deformities.^3,7^ No genetic testing was performed in the presented cases of MS and MAS.

Classic radiographic features of FD are intramedullary, expansile lesions with well-defined borders and endosteal scalloping. The cortex may be thinned but remains intact. Lesions may be lucent, sclerotic, mixed or of ground glass attenuation depending on the age of the patient. Monostotic disease typically involves the rib (28%), femur (23%), tibia, mandible, skull or humerus. Polyostotic disease has a predilection for the femur (91%) as shown in the presented cases, but may also involve the tibia, pelvis, skull and facial bones.

CT is best used to detect the true extent of the lesion and is the modality of choice in craniofacial disease. CT imaging findings include ground glass attenuation, cystic or sclerotic appearance, expansion of bone with maintenance of cortical integrity and endosteal scalloping. In the case of MAS, CT was not performed to avoid unnecessary radiation exposure and because the presentation of precocious puberty demanded the exclusion of hypothalamic-pituitary-axis abnormalities.

Imaging with MR is not particularly contributory or diagnostic because of variability in the appearance of the bone lesions, and may mimic more aggressive bone lesions. Lesions are intermediate to low on T1-weighted imaging, variable on T2-weighted imaging and demonstrate heterogenous moderate to avid enhancement.^8,9^ The case of MAS displayed hypointense signal with MRI on all sequences consistent with sclerosis as seen on radiographs. A post-contrast study was not performed in this instance of MAS. This appearance, although atypical, may suggest low cellularity and increased trabeculation of the bony lesion adding to the varying degree of MRI findings. Additional findings of cystic and haemorrhagic change affect the degree of contrast enhancement of FD bone lesions, which can be highly variable and may suggest intrinsic activity of different bone lesions.^10^

Mazabraud syndrome is a very rare syndrome defined by the presence of intramuscular myxomas in a patient known with FD.^10^ The total incidence of MS is not known; however, approximately 100 cases have been reported in the literature up to 2019.^2^ Intramuscular myxomas are benign, slow-growing lesions commonly found within skeletal muscle. The myxomas can range from single to multiple lesions with the lower limbs more commonly affected, as with the presented patient who had multiple soft tissue tumours in the lower limb that showed features typical of myxomas on MRI.^7,11,12^A higher proportion of MS cases, over 60%, occur in females because of a female inclination, while only 30% of cases are male.

The intramuscular myxomas are homogenously hypoattenuating on CT, located close to areas of FD. On MRI, they are hypo- to slightly hyperintense on T1WI and hyperintense on T2WI with a varied pattern of enhancement. Four different patterns of enhancement have been documented, which include^8,9^ peripheral, peripheral and patchy internal (as in the presented case), peripheral and linear internal, and heterogeneous internal. The presence and amount of myxoid tissue together with tumoural cellularity and vascularity gives rise to the different enhancement patterns.^13^ Distinguishing myxomas from other more sinister and malignant myxoid lesions, likemyxoid liposarcoma and myxofibrosarcoma, pose a diagnostic dilemma. The most reliable imaging features to make the distinction consist of a perilesional fat layer seen on T1WI and increased T2WI signal in the surrounding muscle.^14^ Histological diagnosis is recommended where imaging and clinical features suggest a high suspicion for myxoma with either biopsy or excision.^15^

Diffusion-weighted imaging (DWI) is particularly useful in distinguishing the complication of malignant change. Recent studies show that the evaluation of ADC values in DWI is useful to distinguish benign myxomas from myxoid liposarcomas, where higher ADC values support a more benign lesion.^5,9,16^ Nuclear medicine has a limited role in intramuscular myxoma imaging with the majority of findings described on MRI. There is, however, mild to moderate F18-FDG avidity on PET-CT.^11^ The most common complication of surgical excision of the myxomas is recurrence.^9^ Mazabraud syndrome is associated with a higher incidence of malignant transformation to osteosarcoma,^8^ and thus clinical and radiological surveillance is essential.

McCune–Albright syndrome is a rare form of FD and is characterised by a combination of clinical and biochemical findings. Diagnosis of MAS includes the presence of confirmed polyostotic FD, café au lait skin pigmentation and endocrinopathy. Patients with MAS often present with fractures and short stature because of premature closure of the epiphyseal plates and continuous remodelling of occult fractures. Multiple skeletal abnormalities include leg length discrepancies and osseous deformities. Endocrine-related disorders related to MAS include Cushing’s syndrome, pituitary adenomas, thyroid disease and precocious puberty with the latter being the most common in affected females.^17^ The described patient presented with precocious puberty and skeletal findings suggestive of FD. Further investigation revealed that she was known to the endocrinology unit with hormonal disturbances in the absence of pituitary and thyroid disease. She did not have the café au lait skin pigmentation lesions as classically known with MAS and there was no family history of other genetic diseases. The syndrome affects males and females equally with no gender predilection and a prevalence between 1:100 000 and 1:1 000 000, making it a rare occurrence.^18,19^

McCune–Albright syndrome imaging findings are related to the diagnoses of FD. Once a diagnosis of FD is made, further correlation with biochemistry and clinical information is performed. The role of nuclear medicine imaging in MAS is limited with recent studies suggesting the role of PET-CT imaging with radio-labelled phosphodiesterase-4 inhibitor, ^11^C-(*R*)-rolipram, which evaluates the cyclic adenosine monophosphate pathway (cAMP). The cAMP is altered in regions of dysplastic bone, which can be useful in evaluating the burden of disease and localising lesions.^20^ Bone lesions in FD can therefore be evaluated for disease activity with Technetium 99m-methyldiphosphonate and malignant sarcomatous change can likely be assessed with 18-F-fluorodeoxyglucose; however, nuclear medicine imaging cannot be used primarily to assess for malignancy.^10^

The presented patient with MS underwent an excisional biopsy, which was both therapeutic and diagnostic. Another treatment option for symptomatic myxomas is surgical excision if they cause pain, pressure or neurological symptoms.^21^ The patient with MAS has not undergone any surgical treatment thus far. No medical treatment has thus far been instituted and studies suggest that no clear treatment is yet known for MS with treatment mostly being symptomatic. Medical treatment with anti-resorptive medication, for example bisphosphonates, may relieve pain and reconstitute the lesions with normal bone.^9^ Orthopaedic intervention is generally required for secondary bony fractures, deformities and severe pain.

## Conclusion

Fibrous dysplasia, Mazabraud and MAS are rare, skeletal non-inherited conditions, which present with a spectrum of symptoms ranging from pain and deformity to soft tissue masses. Further endocrine work-up is imperative in patients with FD presenting with café au lait spots and short stature to assess for endocrine abnormalities in the setting of MAS. Similarly, a palpable soft tissue mass may represent a myxoma in the setting of MS, which, although rare, has a relative increased risk of malignant transformation. A thourough understanding of the associated syndromes of FD and their clinical and radiological presentation is crucial to make an accurate and complete diagnosis.

Radiographic findings are typical with bowing deformities, sclerotic, lucent or mixed lesions and bony expansion often with endosteal scalloping. MRI is often non-contributory and may mimic a more aggressive process. Early detection and correct diagnosis allow for early preventative treatment and rehabilitation to prevent devastating neurological sequelae and disability.
